# The Path from Personality to Anxiety and Depression Is Mediated by Cognition in Multiple Sclerosis

**DOI:** 10.3390/jpm14070682

**Published:** 2024-06-25

**Authors:** Alina Schenk, Cosmin Octavian Popa, Cristiana Manuela Cojocaru, Ștefan Marian, Smaranda Maier, Rodica Bălașa

**Affiliations:** 1 The Doctoral School of George Emil Palade, University of Medicine, Pharmacy, Science and Technology, 540142 Targu-Mures, Romania; alina.muresan@umfst.ro (A.S.); cristiana-manuela.cojocaru@umfst.ro (C.M.C.); 2 Department of Ethics and Social Science, George Emil Palade University of Medicine, Pharmacy, Science and Technology, 540142 Targu-Mures, Romania; 3 Department of Psychology, West University of Timişoara, 4 Vasile Pâvan Boulevard, 300223 Timişoara, Romania; stefan.marian@e-uvt.ro; 4 Neurology Clinic I, Emergency Clinical County Hospital, 40136 Targu Mures, Romania; smaranda.maier@umfst.ro (S.M.); rodica.balasa@umfst.ro (R.B.); 5 Department of Neurology, University of Medicine, Pharmacy, Science and Technology, 540142 Targu Mures, Romania

**Keywords:** multiple sclerosis, personality, depression, anxiety, dysfunctional psychological mechanisms, fatigue, health status

## Abstract

Background: Multiple sclerosis (MS) is a neurodegenerative immunological disease causing significant impairment in all life areas. Therefore, personality changes are observed and associated with higher prevalence of depression and anxiety disorders. Considering this relationship, we hypothesized that clinical symptoms and personality disorders are more prevalent in MS and that dysfunctional psychological mechanisms mediate the path from personality disorders to clinical symptoms. Methods: The study sample consisted of 43 patients with MS (age M = 41.9, SD = 11.5) and 31 controls (age M = 39.8, SD = 10.3). Measures of personality, anxiety, depression, fatigue, health status, and dysfunctional psychological mechanisms were conducted. Results: The prevalence of clinical symptoms was increased in MS patients as compared to controls. Also, dependent and schizoid personality traits (PTs) were observed in the patient sample. Negative automatic thoughts (NATs) were found to mediate the association between dependent PT and clinical symptoms. Along with schizoid PT, all dysfunctional psychological mechanisms impacted clinical symptoms. Discussion: The results of our research are in line with previous studies showing that anxiety, depression, and dysfunctional personality traits are more prevalent in MS as compared to controls. Conclusions: PTs and dysfunctional psychological mechanisms predicted depression, anxiety, fatigue, and health status in MS patients. Cognition acts as a strong mediator between PTs and psychopathology in MS. Hence, integrative personalized psychological treatment is recommended to improve the quality of care in MS.

## 1. Introduction

Multiple sclerosis (MS) is a neurological autoimmune inflammatory disease. From a physiological point of view, this condition affects the myelin sheath, which prevents the conduction and transmission of nerve impulses, hindering the entire function of the central nervous system. Although not fully elucidated yet, the causes of the disease can be explained through the interaction of certain environmental and genetic factors [1]. The most frequent symptoms are sensorial, visual, and walking disturbances, problematic intestinal transit and urinary functioning, repeated infections, and cognitive deterioration, as well as social and psychological dysfunctions. Among all of these symptoms, fatigue is perceived as one of the most debilitating [2]. The global prevalence of MS is 35.9 per 100,000, while the prevalence within the Romanian population is 34.8/100,000 [3,4].

The disease course is unpredictable. Over time, it may evolve from relapsing-remitting MS (RRMS), characterized by a stable course and no progression between relapses, either with complete recovery or some remaining deficits after an episode, toward secondary progressive MS (SPMS), defined by the presence of disease progression with or without relapses, or toward the primary progressive MS (PPMS) type, which is less common when the disease progression starts from symptom onset [5].

Even though the disease is currently not curable, there has been a remarkable advance in the pursuit of the best therapeutic directions to slow down its evolution. Therefore, disease-modifying therapies (DMTs), like interferon beta, glatiramer acetate, dimethyl fumarate, natalizumab, and mitoxantrone, and experimental ones have proven to be effective in treating patients with MS, especially RRMS [6]. However, DMT treatment is not without side effects, and longer periods of drug administration frequently correlate with an increased risk of infection [7].

Within the clinical picture of MS, dysfunctional personality traits and personality disorders (PDs) represent stable factors contributing to the development of specific symptoms, such as anxiety and depression. PDs can be defined as pervasive, inflexible, and stable maladaptive patterns of thinking, feeling, and behaving that interfere with the functioning of the person, within a certain socio-cultural context [8]. The Five Factor Model (FFM) represents a common paradigm used to explain several dysfunctional personality traits describing PDs according to the Diagnostic and Statistical Manual of Mental Disorders, Fifth Edition (DSM-5). This model of personality includes five central traits, specifically, neuroticism, extraversion, openness, conscientiousness, and agreeableness [9]. Neuroticism is characterized by an intense reactivity in stressful situations, generating negative emotions and maintaining a constant perception of danger and inability to deal with difficulties [10]. Negative emotionality constitutes the maladaptive dimension proposed by the DSM-5 as an alternative to neuroticism, encompassing multiple dysfunctional traits like anxiety, emotional liability, submissiveness, anhedonia, and separation anxiety. Furthermore, negative emotionality could be perceived as a feature of neuroticism predominately present in anxiety and depression [11]. Extraversion is a hallmark of exuberant, sociable, and dynamic people [8]. The pathological expression of low extraversion is represented by the detachment dimension, which refers to a set of traits like social withdrawal, avoidance of closeness, depressive features, and suspiciousness. Openness refers to the necessity of exploration, intellectual interests, and ingenuity, whereas psychoticism, as its clinical correspondent, includes magical, eccentric thinking [12]. Conscientiousness describes disciplined and organized individuals with a high sense of duty and a strong need of professional achievement. The DSM-5 alternative of this trait is represented by disinhibition, which is composed by impulsivity, risk taking, lack of responsibility, and distractibility. Agreeableness is specific to tolerant, empathic, cooperative persons, while antagonism, the expression of its opposite, refers to hostility, manipulation, duplicity, attention seeking, and grandiose tendencies.

As far as it is known, PDs are strongly related to chronic medical conditions, including MS [13]. Thus, personality changes are often observed in MS patients, with research on this topic revealing that patients are more socially withdrawn, apathic, emotionally unstable, and impulsive [14]. Also, high levels of neuroticism and lower levels of extraversion, consciousnesses, and agreeableness were linked to higher levels of depression and anxiety disorders, fatigue, disability, cognitive impairment, and lower quality of life in MS [15].

Furthermore, the presence of anxiety and depression was found to be higher among people with PDs [16,17]. The co-occurrence of these psychopathologies is highly prevalent in MS patients, with estimates showing that 30–40% [18] of patients suffer from major depressive disorder and 36% [19] from anxiety disorders throughout the course of the disease. Hence, findings emphasized a significant association between psychological symptoms and personality disorders in MS patients.

Particular attention is given to major depressive disorder (MDD) diagnosis in comorbidity with MS. According to the DSM-5, MDD implies that the patient must experience at least five of the following nine specific symptoms for a period of at least two weeks: intense sadness, loss of interest or pleasure, attention and memory deficits, feelings of guilt and worthlessness, suicidal thoughts, insomnia, extreme fatigue or lack of energy, daily psychomotor agitation or retardation, and loss of appetite, with a significant negative impact on individual functionality in all life areas [20].

Alongside MDD, generalized anxiety disorder (GAD) refers to one of the most common types of anxiety disorders found in MS [21]. Specific symptoms are excessive, uncontrollable worries, intense fear, restlessness, irritability, attention and concentration difficulties, fatigue, muscle tension, and insomnia [22]

The onset of psychopathology is studied based on the relationship between personality and cognitive styles [23]. Thereby, according to cognitive theory, dysfunctional schemas represent the core of PDs. Thus, the occurrence of maladaptive responses in stressful situations/negative life events results from the interplay between genetic and environmental factors, which consequently contributes to the development of dysfunctional schemas during childhood, altering one’s perception, attribution, and signification of the present reality [23]. In this way, negative automatic thoughts (NATs), dysfunctional attitudes (DAs), and irrational beliefs (IBs) represent common dysfunctional psychological mechanisms linked to the development of depressive and anxiety disorders in relation to personality and negative life events [24,25]. NATs (e.g., “I am a failure,” “I will never succeed”) could be described as both “cold and hot cognitions,” representing the deeper level of thoughts involved in the evaluation of our representations, which is directly accountable for the occurrence of our emotional, behavioral, and physiological dysfunctional responses in a specific negative or ambiguous situation [26]. DAs represent the most profound level of thinking and consist of strong beliefs about the self and world, developed early in one’s life (e.g., “I am an inferior person because I do not perform as well as others,” “Before starting an activity, you must be sure you can finish it”). These beliefs are challenging within the clinical psychological practice, and the development of a more functional attitude when dealing with uncertainty and negative life situations requires their proper identification and modification [27]. IBs are described as easily accessible thoughts situated at the surface level of thinking, defining one’s representation of relevant events that activate his/her negative emotions. These beliefs are divided into four subtypes, such as demandingness (e.g., “People must be honest!”), self-downing/global evaluation (e.g., “I am a horrible person”), low frustration tolerance (e.g., “I can’t stand to be seek”), and awfulizing/catastrophizing (e.g., “It’s awful to be a single parent”) [28].

Fatigue as a frequent symptom in MS could also be influenced by personality. In this regard, high levels of neuroticism are linked to increased levels of psychological fatigue, whereas lower levels of extraversion are positively correlated with physical fatigue [29]. Additionally, higher fatigue scores were identified in MS patients with depressive, avoidant, and masochistic personality traits [30].

Moreover, the state-of-art in MS states that deficits at the levels of attention, language, memory, perception, orientation, and reasoning are associated with dysfunctional personality traits and psychopathology. Thus, neuroticism and lower extraversion level were correlated with decreased memory functioning [31], while consciousnesses predicted cognitive dysfunctions in MS patients [32]. Additionally, anxious and depressed MS patients were found to have worse performance in objective assessments of cognitive domains [33]. The consequences of the interplay between personality, psychopathology, and cognitive dysfunction are seen in poorer social interactions and quality of life, lower employment rates, and a lack of financial autonomy, further affecting proactive attitudes in MS disease management.

Associated with MS, PDs, depression, anxiety, and fatigue are positively correlated with poor treatment adherence, cognitive impairment, social isolation, suicidal attempts, early retirement, disease symptoms exacerbation, and lower overall quality of life [34,35,36]. To date, several personalized approaches for evaluating the severity of clinical implications in MS, along with individualized treatment protocols addressing different intensity categories, have been proposed. For example, Buja et al. identified specific socio-demographic and psychological variables associated with treatment adherence in MS [37]. Despite that there is salient evidence of the efficiency of psychological interventions for treating depression and anxiety in MS patients, the obtained effect sizes are mild to moderate because of their lack of specificity for this condition [38]. Regarding the individualization of MS management, Kiropoulos et al. proposed a protocol including cognitive and behavioral techniques tailored to address the particularities of the most common MS symptoms, as well as depression and related comorbidities [39].

In this way, identifying the nature of the relationship between anxiety, depression, fatigue, perceived health status (i.e., named symptoms as follows), dysfunctional psychological mechanisms, and personality, particularly PDs, could streamline the multidisciplinary personalized therapeutic approach of the most frequent mental health disorders associated with MS, improving the health-related quality of life. Therefore, the main objective of this study is to investigate the relationship between personality, dysfunctional psychological mechanisms, and symptoms in a clinical sample of Romanian patients diagnosed with MS and a control sample. Thus, the first hypothesis states that the dysfunctional psychological mechanisms and symptom levels are higher in the clinical sample when compared to controls. The second hypothesis affirms that pathological personality dimensions and PDs are more frequent in the clinical sample than controls. Finally, the third hypothesis claims that dysfunctional psychological mechanisms mediate the relationship between personality disorders and symptoms in the clinical sample.

As far as we know, this is the first study that analyses the relationship between PDs, dysfunctional psychological mechanisms, and clinical symptoms in the Romanian MS population.

## 2. Materials and Methods

### 2.1. Participants

G-Power software, version 3.1., was used for calculating the sample size. The input indicators were the following: linear multiple regression statistical test with a fixed model and single regression coefficient for five predictors, moderate effect size (f2 = 0.20), alpha error probability of 0.05, and power of 0.80. This study included 43 patients with MS (31 females and 12 males; mean age: 41.9 years) and 31 controls (25 females and 6 males; mean age: 39.8 years). The patients enrolled in the study were recruited from the database of the Neurological Clinic of the County Emergency Clinical Hospital in Targu Mures. Regarding the patient sample, the inclusion criteria were: (1) a final diagnosis of MS established by a neurologist according to McDonald criteria; (2) minimum age of 18 years or over; (3) undergoing a DMT for treating clinical manifestation of the disease, reducing relapses, and slowing disability progression; and (4) Romanian language speaker. The exclusion criteria were: (1) acute relapsing episode that implies a significant functional impairment requiring a specific treatment intervention; (2) moderate or severe cognitive impairment, including executive function, attention, memory, language, orientation, and abstraction; and (3) diagnosis of severe psychiatric disorders, such as psychotic disorders, schizophrenia, and bipolar disorder. Participants in the control group were enrolled in this study through online invitations. The inclusion criteria for controls were: (1) minimum age of 18 years or over; (2) without diagnoses from the neurological and emotional spectrum; (3) Romanian language speaker; and (4) ability to access technological devices. Prior to the study, written informed consent was obtained from all participants. Also, the study received ethical approval issued by the Ethics Commission of Scientific Research of George Emil Palade University of Medicine, Pharmacy, Science and Technology from Targu Mures, under number 1446 on 22 July 2021.

### 2.2. Measures

The Personality Clinical Form (PCF) [40] is an instrument that was exclusively validated within the Romanian population for the assessment of personality using both categorical and dimensional models. The instrument contains 200 items with dichotomous answers (true or false, scored 1 or 0) and includes over 50 scales, of which 7 are applied for the protocol validation, 26 are single-dimensional scales that evaluate pathological personality traits, 5 scale assess personality dimensions, 3 scales evaluate major psychopathological trends, 10 scales address the classical personality disorders, and the last 4 scales evaluate the functional impairment severity. Based on the resulting scores, two types of profiles (i.e., economical and clinical) are generated through the web app. The instrument has good internal consistency for all 50 scales, with a Cronbach’s alpha between 0.62 and 0.96.

The Beck Depression Inventory II (BDI-II) [41] is a self-assessment tool containing 21 items for evaluating the severity of the major depressive episode symptoms. Each item is rated on a scale from 0 to 3 (0 = low intensity, 3 = high intensity), summing up a maximum score of 63 points. The intensity of depression is given by the total score, which can be reported as follows: 0–9 minimum depression; 10–18 moderate depression; 19–29 major depression, and 30–36 severe depression. We used the Romanian adaptation of the instrument, which presents a Cronbach’s alpha level of 0.90 [42].

The Hamilton Rating Scale for Anxiety (HRAS) [43] is a clinical interview that assesses anxiety severity. From the total of 14 items, 6 items evaluate psychic anxiety (tension, fear, insomnia, concentration difficulties, and depression) and 8 relate to somatic anxiety (cardiovascular, respiratory, gastrointestinal, and autonomic symptoms). Each item is scored by the clinician using a Likert scale from 0 = lack of anxiety to 4 = severe anxiety, with a total score range of 56, where a score of 17 or less indicates mild anxiety, a score from 18–24 indicates mild to moderate anxiety, and a score over 25 indicates moderate to severe anxiety. The Romanian scale has high reliability, indicating a good internal consistency through a Cronbach’s alpha level of 0.92 [44].

The Automatic Thoughts Questionnaire (ATQ) [45] is a self-report questionnaire comprising 15 items, answered on a 5-point scale (from 1 = never to 5 = all the time) for measuring the frequency of negative self-statements or thoughts like: “I’ll never make it” or “I can’t finish anything.” The total score consists of the sum of all items. Higher scores show an increased frequency of negative automatic thoughts. The Romanian version used in this research has a high internal consistency with a Cronbach’s alpha coefficient of 0.92 [46].

The Dysfunctional Attitudes Scale -A (DAS-A) [47] is a self-report scale composed of 40 items that assess the intensity of maladaptive beliefs and cognitive distortions on a 7-point Likert scale (from 7 = totally agree to 1 = totally disagree). Each item includes statements as: “If others dislike you, you cannot be happy” or “If I do not do well all the time, people will not respect me.” Ten items are reversely coded, with a total score involving the sum of all items. Higher scores represent the presence of multiple intense dysfunctional attitudes. We used the Romanian version of the scale, which shows good psychometric properties [48].

The Attitudes and Belief Scale, Second Edition (ABS II) [49] is a self-reported scale that measures irrational and rationales beliefs at all four cognitive levels: demandingness (i.e., “I must be liked by the people I consider important for me, and I will not accept not being liked by them.”), self-downing (i.e., “I think I’d be a worthless person if I got poor results on important tasks for me.”), low frustration tolerance (i.e., “I can’t bear to be tense or nervous and I think these states are unbearable”), and awfulizing (i.e., “It is terrible to have a quarrel in a man’s life, and it is a disaster to be upset with someone.”). The scale includes 72 items rated on a 5-point Likert scale (0 = strongly disagree and 5 = strongly agree). The higher the total score, the greater is the irrationality. The Romanian version used in this study has good reliability, with an alpha Cronbach’s of 0.87 for the total score [50].

The Chalder Fatigue Scale (CFS) [51] is a self-reported instrument that evaluates fatigue based on 11 items, with the first seven items measuring physical fatigue (i.e., “Do you need to rest more?”), while the last four evaluate cognitive fatigue (i.e., “Do you have difficulties concentrating?”). Each item can be scored using a 4-point Likert scale from 0 to 3 (0 = better than usual, 1 = no worse than usual, 2 = worse than usual, and 3 = much worse than usual). The higher the total scores, the greater the fatigue. The scale has good internal consistency, with Cronbach alpha levels ranging between 0.86 and 0.92 [51].

The EQ-5D-3L [52] is a standardized self-reported measure of health status comprising 5 dimensions assessed using a 3-point Likert scale (1 = no problems, 2 = moderate problems, 3 = severe problems). These five domains include mobility, self-care, usual activities, pain/discomfort, and anxiety/depression. The instrument demonstrates high internal consistency, valid redistribution, and good construct validity [53].

### 2.3. Design and Procedure

The present study was carried out between 2021 and 2023 and was designed as an observational study including two convenience samples of participants, namely, a clinical group and a control group. By accessing the clinic’s database, a member of the research team completed the first phase of the procedure selecting patients eligible for the present study. In this way, information related to the diagnosis, treatment, and disability level was selected, including data regarding the cognitive impairment severity. After this stage, selected patients were telephonically contacted and the research project was presented in detail, planning the screening for the interested patients on the same day as the medical treatment. Before starting the assessment, each participant enrolled in the study signed the informed consent form. The research protocol included the PCF for personality assessment, the BDI-II, HRAS, CFS, and EQ-5D-3L for evaluating clinical symptoms, along with the ATQ, NATs, and ABS II for the assessment of dysfunctional psychological mechanisms. The anxiety interview was conducted by a clinical psychologist. After the evaluation ended, the results were included in the research database. Participants in the control group were invited to fill in the forms, which were provided online, except for HRAS, which was conducted telephonically.

### 2.4. Statistical Analysis

Statistical analysis was performed in R version 4.3.2 [54]. For comparisons between the clinical sample and control group, we used Welch’s *t*-test performed using the t test function in R package rstatix [55]. Welch’s *t*-test allows for the comparison of groups that have unequal variances. In cases where the variances of the two groups are equal, Welch’s *t*-test produces results similar to a standard Student’s *t*-test. Therefore, we applied Welch’s *t*-test for all comparisons. To test the normality assumption, we computed skewness and kurtosis overall and separately by group using the skew and kurtosis functions in the package psych [56]. A distribution was considered normal when the skewness value was between −2 and 2 and the kurtosis value was between −7 and 7.

We first performed multiple linear regression between the PDs separately (i.e., schizoid and dependent) and the four outcomes (i.e., HRAS anxiety, BDI-II depression, CFS fatigue, and EQ-5D-3L perceived health status). Due to the particularities of our sample, we also included six other predictors that we considered to be covariates. These predictors included age, disability, and disease duration, which were continuous variables, and three more dichotomous variables, namely gender (1 = male, 2 = female), occupation (1 = unemployed, 2 = employed), and education (1 = low level, 2 = high level). The last two had to be recoded as they were measured on a nominal scale and due to low frequencies for some levels of these variables. For occupation, those that answered “housewife,” “retired,” or “student,” were coded as “unemployed,” and only those who indicated “employed” were coded as such. For education, participants who indicated that they had “higher education,” “post-high school,” and “professional studies” were coded as “high level,” while “high school” and “middle School” were coded as “low level.”

**Figure 1:**
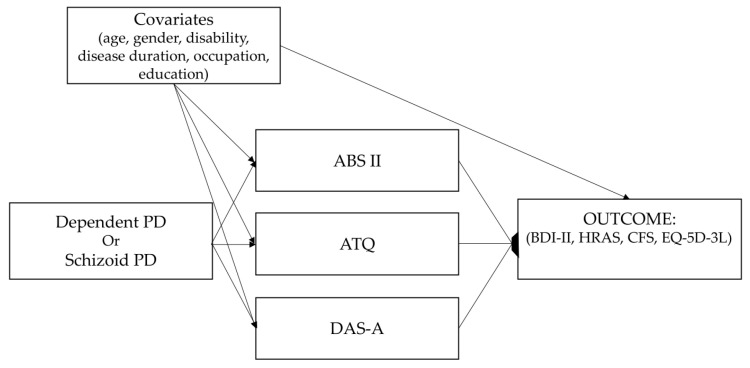
Hypothetical mediation model.

To test the mediation effect of dysfunctional psychological mechanisms on the relationship between personality disorder and outcome variables, we estimated eight mediation models using manifest variables—one for each of the two PDs and combined with one of the four dependent variables (i.e., anxiety, depression, fatigue, and health status). Thus, each model included: (1) one personality disorder; (2) three mediators; and (3) one dependent variable at a time. To further control for the particularities of the sample and obtained unbiased estimates, we used the same covariates as in the multiple regression models. The hypothetical mediation model applied to each variable is presented in Figure 1. The mediation analyses were performed using the package *lavaan* version 0.6 [57] in R version 4.3.3 [54].

## 3. Results

Our study included 43 patients in the clinical sample with a mean age of 41.9 (SD 11.5), among whom 72.1% were female, 67.4% were married, and 46.5% were retired. The levels of disability using the Expanded Disability Status Score (EDSS) were <4.5 for 76.74% of the patients and >5 but not higher than 7 for 23.25%. The RRMS type was predominant for 93.02%, while 4.65% of patients were diagnosed with the PPMS type and 2.32% with the SPMS type. The average disease duration in years was 10.14 (SD 7.26). There were no significant differences between the clinical sample and control group, except for the educational and occupational levels. Specifically, subjects from the control group had higher educational levels, with 90.3% declaring a higher education level, as compared with 46.5% in the clinical sample. Regarding occupational level, 83.9% of the control group were employed, in comparison with 39.5% in the clinical sample. Table 1 comprises the demographic characteristics of the study groups. 

**Figure 2:**
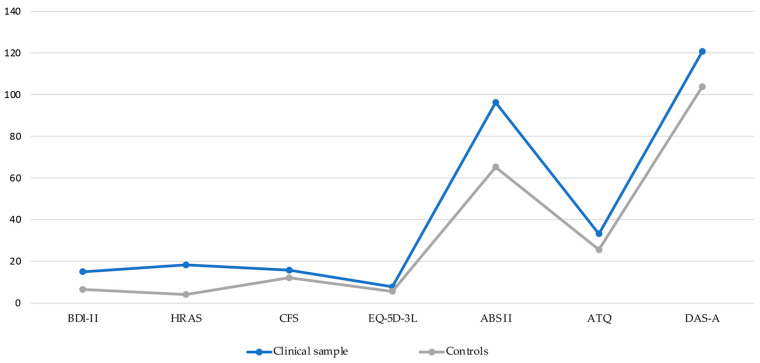
Between-group comparisons for clinical symptoms and psychological dysfunctional mechanisms.

The results of comparisons between the two groups are presented for anxiety, depression, fatigue, health status, and dysfunctional psychological mechanisms are presented in Table 2. The table presents group means and standard deviations together with results from Welch’s *t*-test and Cohen’s d measure for effect size. All data presented had a normal distribution, falling in the −2–+2 interval for skewness and −7–+7 for kurtosis. In general, the clinical sample had larger means than the controls for depression (t = −4.05, *p* = 0.00, d = 0.91), anxiety (t = −6.34, *p* = 0.00, d = 1.45), NATs (t = −2.64, *p* = 0.01, d = 0.61), IBs (t = −2.91, *p* = 0.00, d = 0.67), fatigue (t = −2.44, *p* = 0.02, d = 0.55), and health status (t = −6.38, *p* = 0.00, d = 1.42), with the exception of DAs, which was only marginally significant (t = −1.96, *p* = 0.05, d = 0.45). Between-group comparisons are presented in graphical form in Figure 2.

**Figure 3:**
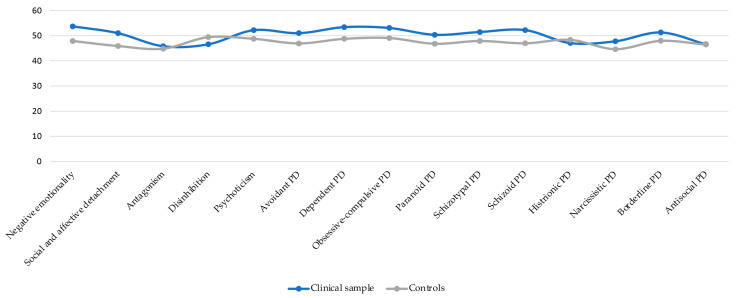
Between-group comparisons for personality dimensions and personality disorders.

In Table 3 are presented comparisons of PCF pathological personality dimensions and accentuated personality traits between the clinical sample and controls using Welch’s *t*-test. The variables were normally distributed in both groups. The two groups were different only in some personality dimensions, with the majority of results being unsignificant. Thus, in the clinical sample, negative emotionality (t = −2.54, *p* = 0.01, d = 0.60) and social and affective detachment as personality dimensions (t = −2.64, *p* = 0.01, d = 0.61) scored higher. As for personality disorders, dependent (t = −2.15, *p* = 0.04, d = 0.51) and schizoid (t = −2.64, *p* = 0.01, d = 0.61) personality traits (PTs) were observed in comparison with controls. Between-group comparison are presented in graphical form in Figure 3.

### Multiple Regression and Mediation

Descriptive statistics and the correlation matrix of all of the variables included in the mediation analyses can be found in Table 2 and Table 3. Every variable included in the analysis presented a normal distribution (−2 < skewness < 2 and −7 < kurtosis < 7).

The results of simple linear regression analyses are presented in Table 4. These results indicated direct relationships between accentuated personality traits and outcome dependent variables. The scores for both schizoid and dependent PTs were significantly associated with all dependent variables.

The results of multiple linear regression analyses are presented in Table 4. These results indicated direct relationships between PTs and outcome clinical symptom variables. These coefficients were extracted from the multiple regressions models that included predictors that we treated as covariates. Therefore, the presented regression coefficients are corrected for the influence of covariates. The scores for both schizoid and dependent PTs were significantly associated with all clinical symptoms.

Regarding covariates, most of the relationships were unsignificant. For the sake of brevity, we report only significant results. Namely, the results of multiple regression indicated that EDSS significantly predicted perceived health status in both models: in the model for dependent PTs, B = 0.55 [95% CI 0.20, 0.89], β = 0.46, t(35) = 3.21, *p* = 0.003, *p* < 0.05, and in the model for schizoid PTs, B = 0.57 [95% CI 0.19, 0.94], β = 0.47, t(35) = 3.07, *p* = 0.004, *p* < 0.05. These results indicated that participants with higher levels of disability reported lower levels of perceived health status.

In Table 5 and Table 6 are presented the results of the mediation analysis. Specifically, Table 5 presents the coefficients of all direct paths and Table 6 presents the indirect paths for each mediator, the total indirect effects, and the total effect. The direct effects that are presented in the table refer to the effect of PTs on the outcome when taking into account the indirect effect through the mediators. The total indirect effect refers to the effect of all three mediators taken together, while the total effect refers to the effect of the three mediators and the direct relationship between the predictor (i.e., PTs) and outcome variable. NATs seemed to predominantly act as a mediator between dependent PTs and clinical symptoms. Thus, NATs fully mediated the relationship between dependent PTs and depression, with z = 2.22, *p* = 0.03, 95% CI (0.03, 0.43), and anxiety, with z = 2.11, *p* = 0.01, 95% CI (0.01, 0.38). Additionally, NATs partially mediated the relationship between dependent PTs and fatigue, with z = 2.31, *p* = 0.02, 95% CI (0.023 0.36), and health status, with z = 2.29, *p* = 0.02, 95% CI (0.00,0.08). Dependent PTs and schizoid PTs, in relation to the effects of NATs, Das, and IR, impacted all clinical variables, except for schizoid PTs on health status.

## 4. Discussion

Overall, the findings of this study showed heightened levels of maladaptive personality traits and dysfunctional psychological mechanisms in the clinical sample, in contrast to controls, highlighting that the relationship between PDs and common MS symptoms is intermediated by dysfunctional psychological mechanisms. First, as compared to controls, the clinical sample presented higher levels of anxiety, depression, fatigue, and poorer perceived health condition. This is concordant with previous research on the prevalence of clinical symptoms in this population. For example, after including more than 70 studies in their systematic review and meta-analysis, Boechoten et al. concluded that the annual prevalence of MDD was 17% in the MS population, as compared to GP (2–10%), while the prevalence of GAD was 14% [58]. The same results were reported by Peres et al. in a recent meta-analysis, where the prevalence of depressive and anxiety clinical symptoms was 27.01% and 35.19%, respectively, establishing a direct link to the impairment of motor functioning [59]. In a similar way as in our study, higher fatigue levels were associated with a significantly lower quality of life [60]. With regard to perceived health status in the clinical sample, our outcome is in line with numerous studies emphasizing the burden of neurological, psychological, and physiological symptoms on overall quality of life [61,62].

In addition, at a cognitive level, dysfunctional psychological mechanisms, except for DAs, were more frequent in the clinical sample, underlining the proneness of these patients to present distorted thinking styles. In this way, the presence of these dysfunctional psychological mechanisms contributes to the onset of psychopathology. This is in line with other studies exploring the role of cognitive style in the development of depressive and anxiety symptoms in association with MS. Similar to our results, Güner et al. pointed out the higher frequency of NATs but not DAs in MS patients, in contrast to controls [63]. The absence of statistically significant differences in DAs between groups could be justified by the predictive role of this type of dysfunctional thinking in relation to psychopathology, in contrast to the proximal role of NATs in the onset of anxiety and depression [64,65]. Also, Văcăraș et al. identified an enhanced NAT frequency in a sample of Romanian MS patients with clinical symptoms of anxiety and depression [66]. In addition, high IB scores were observed in clinical psychopathology samples, as opposed to the sub-clinical group [67].

Second, from the dimensional perspective of personality functioning, elevated negative emotionality along with social and affective detachment traits were identified in our clinical sample. This means that patients with negative emotionality are prone to respond to stressful situations with intense negative emotions caused by a set of negative dysfunctional beliefs about the self, others, and the world. This dimension predisposes patients to develop health problems and recurring depression and anxiety episodes. The high scores of social and affective detachment traits in our clinical sample, as compared to controls, indicate the tendency of patients to avoid social interaction and to evade intimate relationships, experiencing intense levels of anxiety when constrained to face social contexts [68]. In the same light, the tendency toward negative affect was found to be a prominent trait in MS by other researchers. For example, assessing the relationship between personality traits and emotional disorders, Vaheb et al. discovered that neuroticism positively correlates with psychopathology. In addition, high levels of neuroticism were also associated with lower levels of extraversion, agreeableness, and consciousnesses [69]. In the same direction, Chu et al. observed an increased level of neuroticism in their MS studied sample and lower levels of extraversion, both correlating with the presence of anxiety and depression [70]. Therefore, due to this personality profile, MS patients are more likely to experience increased anxiety, depression, anhedonia, social withdrawal, avoidance of closeness, and submission, in comparison with controls.

Employing a categorial perspective of personality using the Million Clinical Multiaxial Inventory (MCMI-II), Mirzaei et al. revealed that MS patients presented avoidant, passive-aggressive, borderline personality patterns, anxiety, depression, thought disorders, and somatization for clinical syndromes [71]. Also, PD assessment using the Structured Clinical Interview for DSM (SCID-II) indicated that avoidant, obsessive-compulsive PD were more frequently observed in MS patients, in comparison with controls [72]. The evaluation of personality clinical features in our clinical sample indicated the presence of dependent and schizoid PTs. Thus, based on the peculiarities of these PTs, it was expected that some of the patients would experience increased levels of anxiety due to their trouble undertaking daily decisions and their constant need to be in the care of another person, while others would be more socially and emotionally isolated. Among other dependent PT characteristics, insecurity in expressing their disagreement and lack of self-confidence in completing an activity without needing the help and guidance of others were outstanding in MS patients. Moreover, they were determined to have a submissive attitude toward others to avoid rejection. As a result of this dysfunctional trait, when a romantic relationship ends, they quickly sought to start another relationship driven by the of fear of not losing their “compass” [73]. Another part of the patients in our investigation presented schizoid PTs, showing detachment from social interaction and reduced emotional expression [74]. They tended to be indifferent to others’ remarks, living in solitude, and showing no interest in romantic relations. All of these specific traits were reinforced by their disability, correlating with progression of the disease, as well as the personal coping style used to overcome MS general symptomatology. The results of our study, together with those of previous research, reinforce the evidence of increased prevalence of PTs in MS, as opposed to controls [72]. This is concordant with the theory of negative emotionality as a common feature linking dysfunctional personality and psychopathology [11]. The presence of PDs negatively impacts the patient’s disease management and worsens its clinical manifestation, reducing the use of adaptative coping strategies and intensifying the frequency of affective and anxiety disorders, together with their psychological mechanisms.

Although the relationship between PDs and MS has been established, it is not yet possible to point to the cause of their co-occurrence. Therefore, changes in personality could be explained through the interplay of different determinants, such as specific brain dysfunctions, adverse effect of DMTs, psychological and social factors, pre-existing psychopathology, personality traits, and cognitive styles [14]. Further studies could explore the etiology of PD occurrence within the MS population in more detail, generating multiple treatment alternatives for alleviating the burden of living with this illness.

When including the confounds, only health status was influenced by disability for both PTs, meaning that patients having higher disability levels reported lower perceived health status. The impact of disability on the relationship between dysfunctional PTs and perceived health status, as well as its implications for overall quality of life was also pointed out in previous studies [75]

Also, in contrast to controls, the clinical sample presented lower educational levels and higher levels of retirement. However, these sociodemographic factors did not relate to the relationship between personality and clinical symptoms within our clinical sample. This is opposed to previous research highlighting the protective role of educational achievement for the development of mental health difficulties in adulthood. Thus, the likelihood of psychiatric disorders and substance use in individuals with a lower educational level was higher than in those who were highly educated [76]. Concerning occupational status, professionally active patients reported less mental health problems and a better quality of life [77]

The third hypothesis and the major finding of our investigation was that NATs significantly mediated the impact of dependent PTs on clinical symptoms. This is in accordance with other conclusions stating that NATs are important underlying mechanisms involved in PDs and mental health disorders, as proven within the cognitive theory of psychopathology. Thus, the impact of child adversity on the development of PDs and depression proved to be mediated by NATs in a meta-analysis conducted by Zhao et al. [78]. Furthermore, NATs were identified as the primary dysfunctional cognitive mechanisms related to other psychological processes that contribute to the maintenance of mental health disorders [79,80,81].

Also, all three dysfunctional psychological mechanisms, including NATs, in relation with dependent PTs mediated the effect on perceived health status. This composite effect of mediation could be related to the daily stress resulting from disease-specific symptoms, which could further activate the dependent core dysfunctional beliefs of helplessness, distorting the perception of health problems in an amplifying manner. In this way, dealing with self-care and daily activities would require a family member’s help and emotional support for dealing with the discomfort and emotional problems. In addition, dependent persons have the tendency to excessively utilize medical health services due to their multiple somatic complaints, as compared to controls [82]. Interestingly, the interaction between schizoid PTs and all three mechanisms taken together impacted all variables, except perceived health status. This outcome can be attributed to the specific characteristics of schizoid PD. Specifically, their social and emotional detachment as well as preference for seclusion contribute to the enhancement of specific dysfunctional and irrational beliefs related to the self and others triggered when their comfort space is threatened. These dysfunctional attitudes facilitate the occurrence of negative and distorted thinking, leading to maladaptive responses. For example, considering the requirements of disease management, patients are regularly forced to expose themselves in social medical environments and to rely on the others’ help for dealing with MS, which could trigger increased levels of anxiety. Surprisingly, the relationship between personality and depression was mediated by the interaction between all three psychological dysfunctional mechanisms. The cumulative effect of these mechanisms emphasizes the relevance of Beck’s unified model of depression. Thus, the interplay between early aversive events and certain genetic factors generates dysfunctional information processing and heightened stress reactivity Over time, they contribute to the development of depressogenic schemas (i.e., negative cognitive triad: negative beliefs about the self, others, and the future), which in turn strengthen biased information processing and stress reactivity [83]. In the case of MS patients, the depressive schemas could be activated by the dysfunctional processing of disease clinical manifestations and their biological response to these stressful situations, with dysfunctional psychological mechanisms being a core component of dysfunctional informational processing.

Along with these, the interaction between dysfunctional psychological mechanisms and PTs mediated the relationship between both PTs and symptoms. This emphasizes that these cognitive dysfunctional mechanisms potentiate the specific manifestation/expression of PDs in MS patients.

The outcomes of the present study emphasize the value of the biopsychosocial model of neuropsychiatric disorders in MS, specifically, the associations between genetic risk factors, neurobiological factors like brain lesions and MRI modifications, immunological factors, and psychosocial factors such as negative, distorted thinking style, lower self-esteem, feelings of hopelessness, dysfunctional coping strategies, interpersonal relationship difficulties, unemployment, and perception of the lack of social support [84,85]. Therefore, a multimodal and integrative approach of the psychopathology co-occurring with MS would increase patients’ resilience in facing the disease burden.

The main result of our research was demonstrating the mediating role of cognitive style on the relationship between personality and psychopathology on a Romanian sample of MS patients, including the covariates that were found to influence these relationships in previous studies [86]. However, personality modification in MS patients and the presence of depression were both associated with lesions in different brain areas, such as the prefrontal cortex or hippocampus, which are responsible for emotion regulation and inappropriate behaviors [87]. Therefore, neuroendocrinological factors could be considered in conjunction with dysfunctional psychological mechanisms.

**Limitations and future directions**

The interpretation of our results could be hindered by several limitations. First, our sample involved a small number of participants. Therefore, the generalizability of our outcomes is restrained to the specific patient sample included in this study. Hence, the observed effect should be interpreted with caution. Second, most assessment instruments had a self-reported format. This involved a subjective evaluation of symptoms and psychological mechanisms. Also, the assessment of personality using a structured clinical interview conducted by a clinician specialized in psychopathology would provide a more objective perspective on the pervasiveness, dysfunctionality, and persistence of the identified patterns in our sample, as well as the accuracy and reliability of diagnosis. Third, the cross-sectional design used in the present study permitted drawing conclusions on the associations of the included variables at one time point. In this way, upcoming studies could reinforce the mediating effect found in this research by conducting longitudinal assessment of personality traits, clinical symptoms, and dysfunctional psychological mechanism.

Starting from our outcomes emphasizing the mediating role of dysfunctional psychological mechanisms, further investigations could identify other psychological processes that prove to be involved in the path from personality to clinical symptoms [88].

Moreover, for a more comprehensive and tailored treatment of MS patients, considering the variability and complexity of clinical manifestations, future studies using larger sample sizes might explore the associations between neurological, biological, psychological, and environmental factors for explaining the impact of dysfunctional personality traits on both physical and psychological outcomes.

**Clinical implications**

For optimal monitoring and prognostication of disease progression in MS, the use of a multimodal and integrative evaluation of both specific clinical symptoms and personal characteristics represents a priority toward individualized care [89,90]. This study is important because it is for the first time dysfunctional psychological mechanisms have been studied as mediators in the connection between personality and clinical symptoms in MS, considering potential confounding factors. Therefore, inclusion of an exhaustive assessment of personality and clinical symptoms, especially dysfunctional psychological mechanisms found to mediate the relationship between PDs and psychopathology, at disease onset or whenever neurological, physical, and psychological changes are observed, is highly recommended. This would enhance the diagnosis process and the clinical case conceptualization facilitating early access to specialized psychological interventions tailored to the specificity of psychological difficulties associated with the disease manifestation in each MS patient. In addition, following personalized psychological interventions would optimally adjust to personal needs and treatment requirements of MS patients. Given the increased symptomatology heterogeneity in chronic medical conditions, personalized intervention aiming to alleviate salient predictors of psychopathology while improving wellbeing correlates could improve the effect sizes of elective treatment [91]. In this way, we consider that cognitive behavioral therapy (CBT) intervention for PDs [24] could be efficient for decreasing both dysfunctional psychological mechanisms and clinical symptoms in MS. According to Brauer and Reinecke, the protocol for dependent PD includes the next personalized therapeutic cognitive-behavioral strategies: cognitive restructuring, behavioral experiments for reducing NATs and DAs, role play for developing assertive communication skills, relaxation for managing high levels of anxiety, in vitro and in vivo exposure to develop autonomy and interpersonal self-efficacy, as well as affect regulation strategies [92]. Likewise, Renton and Mankiewicz emphasized the importance of collaboration strategies in schizoid PD for increasing patients’ involvement in the therapeutic process [93]. The inclusion of techniques like mindfulness [94] for dealing with worries and ruminations along with behavioral activation for managing fatigue [95] and insomnia prevention strategies [96] would enable adjustment of the described protocol for MS patients. In addition, family and social support would be beneficial for encouraging the implementation of therapeutical gains. Considering that CBT protocols used for decreasing anxiety and depression symptomatology co-occurring with MS rely on 4–12 sessions [97], we consider that a minimum of 16 sessions would be recommended. In this regard, future investigations could test the proper length of the treatment plan needed to achieve positive changes on maladaptive behavioral and emotional patterns. By alleviating psychopathological features in MS, patients’ willingness to comply with medical indications and embrace different treatment options could also be strengthened. Considering the nature of the relationship between these constructs would promote an integrative approach aiming to maximize healthcare quality and general wellbeing in this patient population.

## 5. Conclusions

This study showed that the frequency of anxiety, depression, and dysfunctional personality traits was higher in a clinical sample, as compared to controls. Moreover, dysfunctional psychological mechanisms, particularly NATs, were found to mediate the relationship between dysfunctional personality traits and clinical symptoms. Consequently, the conduction of an inclusive clinical assessment to identify dysfunctional psychological mechanisms for properly diagnosing the psychiatric and somatic comorbidities associated with MS is strongly recommended. Moreover, the elaboration and implementation of a multimodal and integrative CBT protocol targeting cognitive mediation between personality and psychopathology can be considered a personalized therapeutic approach of mental health disorders highly associated with MS.

## Figures and Tables

**Table 1:** Sociodemographic characteristics.

	Clinical Sample (n = 43)		Controls (n = 31)		Overall (n = 74)	
Age (mean|SD)	41.9	11.5	39.8	10.3	41	11
	N	%	N	%	N	%
Sex						
Female	31	72.1	25	80.6	56	75.7
Male	12	27.9	6	19.4	18	24.3
Marital status						
Divorced	3	7	2	6.5	5	6.8
In a relationship	0	0	1	3.2	1	1.4
Married	29	67.4	19	61.3	48	64.9
Single	11	25.6	9	29	20	27
Education						
High school	17	39.5	3	9.7	20	27
Higher education	20	46.5	28	90.3	48	64.9
Middle school	2	4.7	0	0	2	2.7
Post-high school studies	1	2.3	0	0	1	1.4
Professional studies	3	7	0	0	3	4.1
Occupation						
Employed	17	39.5	26	83.9	43	58.1
Retired	20	46.5	1	3.2	21	28.4
Student	4	9.3	4	12.9	8	10.8
Housewife	2	4.7	0	0	2	2.7
Type of MS						
RRMS	40	93				
SPMS	1	2.3				
PPMS	2	4.7				
Disease duration (mean|SD)	10.14	7.26				
EDSS (mean|SD)	3.59	1.69				
≤4.5	33	76.7				
≥5 not >7	10	3.3				

Note. SD = standard deviation, MS = multiple sclerosis, RRMS = Relapsing-Remitting Multiple Sclerosis, SPMS = Secondary Progressive Multiple Sclerosis, PPMS = Primary Progressive Multiple Sclerosis, EDSS = Expanded Disability Status Score.

**Table 2:** Comparisons between clinical sample and controls.

	Clinical Sample (n = 43)		Controls (n = 31)					
Variable	M	SD	M	SD	t	df	*p*	d
HRAS	18.30	11.49	4.13	7.72	−6.34	71.71	0.00	1.45
BDI-II	15.02	11.76	6.55	5.98	−4.05	65.70	0.00	0.91
ABS II	96.21	52.67	65.26	38.92	−2.91	71.94	0.00	0.67
DAS-A	120.67	44.04	103.81	30.01	−1.96	71.82	0.05	0.45
ATQ	33.23	14.24	25.58	10.69	−2.64	71.86	0.01	0.61
CFS	15.79	7.71	12.19	4.98	−2.44	71.25	0.02	0.55
EQ5D3L	7.86	2.03	5.65	0.88	−6.38	60.86	0.00	1.42

Note. M = mean, SD = standard deviation, df = degrees of freedom, d = Cohen’s d; HARS = Hamilton anxiety rating scale, BDI-II = Beck Depression Inventory II, ABS II = Attitudes and beliefs scale, DAS-A = Dysfunctional attitudes scale, ATQ = Automatic thoughts questionnaire, CFS = Chalder fatigue, EQ5D3L = Health status.

**Table 3:** Comparisons on PCF dimension scale and personality disorders.

	Clinical Sample (n = 43)		Controls (n = 31)					
Variable	M	SD	M	SD	t	df	*p*	d
Negative emotionality	53.72	9.40	47.90	9.97	−2.54	62.43	0.01	0.60
Social and affective detachment	51.02	9.08	45.90	7.54	−2.64	70.49	0.01	0.61
Antagonism	45.81	8.97	44.84	10.60	−0.42	57.96	0.68	0.10
Disinhibition	46.58	8.22	49.42	8.98	1.39	61.22	0.17	0.33
Psychoticism	52.19	8.39	48.77	9.59	−1.59	59.36	0.12	0.38
Avoidant PD	51.00	9.08	46.94	9.52	−1.85	62.94	0.07	0.44
Dependent PD	53.40	9.07	48.74	9.26	−2.15	63.99	0.04	0.51
Obsessive-compulsive PD	53.07	11.65	49.06	10.75	−1.53	67.65	0.13	0.36
Paranoid PD	50.37	9.54	46.81	8.83	−1.66	67.55	0.10	0.39
Schizotypal PD	51.47	9.84	47.90	9.48	−1.57	66.18	0.12	0.37
Schizoid PD	52.23	9.40	46.97	7.71	−2.64	70.72	0.01	0.61
Histrionic PD	47.07	6.89	48.35	9.65	0.63	51.15	0.53	0.15
Narcissistic PD	47.77	9.15	44.65	9.20	−1.44	64.58	0.15	0.34
Borderline PD	51.28	8.31	47.94	7.96	−1.75	66.39	0.08	0.41
Antisocial PD	46.58	9.28	46.45	10.56	−0.05	59.51	0.96	0.01

Note. M—mean, SD—standard deviation, t—Welch’s *t*-test, df—degrees of freedom, *p*—statistical significance, d—Cohen’s d, PD-personality disorder.

**Table 4:** Multiple regression results.

Outcome	Intercept				Slope							F(7, 35)	*p*	r_adj_^2^
	B	SE	95% CI		B	SE	95% CI		β	t	*p*			
			LL	UL			LL	UL						
Dependent PT score as predictor														
HARS	−16.42	11.97	−40.73	7.88	0.63	0.18	0.27	0.99	0.5	3.55	0.001	2.96	0.01	0.25
BDI-II	−15.4	13.73	−43.28	12.48	0.53	0.2	0.12	0.94	0.41	2.61	0.01	1.37	0.24	0.06
CFS	−16.57 *	7.75	−32.29	−0.84	0.53	0.11	0.30	0.76	0.62	4.62	<0.001	3.6	0.005	0.30
EQ5D3L	0.72	1.83	−2.99	4.44	0.07	0.03	0.02	0.13	0.31	2.6	0.01	5.7	<0.001	0.44
Schizoid PT score as predictor														
HARS	−12.2	11.51	−35.56	11.17	0.6	0.18	0.24	0.97	0.49	3.34	0.002	2.75	0.02	0.23
BDI-II	−9.71	13.28	−36.67	17.26	0.47	0.21	0.04	0.89	0.37	2.24	0.03	1.1	0.38	0.02
CFS	−8.97	8.06	−25.34	7.40	0.43	0.13	0.17	0.69	0.52	3.41	0.002	2.11	0.07	0.16
EQ5D3L	3.11 *	1.86	−0.67	6.89	0.03	0.03	−0.03	0.09	0.14	1.06	0.30	4.25	0.002	0.35

Note. N = 43; B—unstandardized regression coefficient, SE—standard error, t—t-value *p*—*p*-value, LL—lower limit of confidence interval; UL—upper limit of confidence interval; r_adj_^2^—adjusted r squared, PT—personality trait, * *p* < 0.05.

**Table 5:** Direct paths from the mediation models.

		Dependent PT Score						Schizoid PT Score					
Criteria	Predictor	B	SE	z	*p*	[95% CI]		B	SE	z	*p*	[95% CI]	
						LL	UL					LL	UL
ABS II	PT	1.04	0.80	1.30	0.20	−0.53	2.60	0.52	0.82	0.64	0.52	−1.07	2.12
DAS-A	PT	1.61	0.67	2.41	0.02	0.30	2.92	0.76	0.71	1.08	0.28	−0.62	2.15
ATQ	PT	0.56	0.22	2.50	0.01	0.12	1.00	0.45	0.23	1.93	0.05	−0.01	0.90
HRAS anxiety	PT	0.40	0.15	2.64	0.01	0.10	0.69	0.42	0.14	3.09	0.002	0.16	0.69
	ABS II	0.01	0.03	0.33	0.75	−0.04	0.06	−0.001	0.02	−0.04	0.97	−0.05	0.05
	DAS-A	0.02	0.03	0.49	0.63	−0.04	0.07	0.04	0.03	1.58	0.11	−0.01	0.10
	ATQ	0.36	0.09	3.96	<0.001	0.18	0.53	0.33	0.09	3.83	<0.001	0.16	0.49
BDI-II depression	PT	0.22	0.14	1.55	0.12	−0.06	0.51	0.24	0.13	1.83	0.07	−0.02	0.50
	ABS II	0.06	0.02	2.59	0.01	0.02	0.11	0.06	0.02	2.44	0.02	0.01	0.10
	DAS-A	0.01	0.03	0.23	0.82	−0.05	0.06	0.02	0.03	0.86	0.39	−0.03	0.08
	ATQ	0.41	0.09	4.80	<0.001	0.24	0.58	0.40	0.08	4.77	<0.001	0.23	0.56
CFS fatigue	PT	0.40	0.10	4.19	<0.001	0.21	0.58	0.29	0.10	3.00	0.003	0.10	0.48
	ABS II	−0.01	0.02	−0.56	0.58	−0.04	0.02	−0.02	0.02	−1.33	0.18	−0.06	0.01
	DAS-A	−0.03	0.02	−1.70	0.09	−0.07	0.01	−0.001	0.022	−0.04	0.97	−0.04	0.04
	ATQ	0.34	0.06	6.10	<0.001	0.23	0.45	0.35	0.06	5.80	<0.001	0.23	0.47
EQ-5D-3L health status	PT	0.05	0.02	1.97	0.05	0.00	0.09	−0.00	0.02	−0.02	0.99	−0.04	0.04
	ABS II	0.001	0.004	0.13	0.89	−0.01	0.01	−0.002	0.004	−0.56	0.58	−0.01	0.01
	DAS-A	−0.01	0.01	−2.62	0.01	−0.02	−0.003	−0.01	0.01	−1.75	0.08	−0.02	0.001
	ATQ	0.08	0.01	5.74	<0.001	0.05	0.11	0.09	0.01	6.20	<0.001	0.06	0.11

Note. N = 43; B—unstandardized regression coefficient, SE—standard error, z—z-value, *p*—*p*-value, LL—lower limit of confidence interval; UL—upper limit of confidence interval.

**Table 6:** Indirect paths and total effects.

	Dependent PT Score						Schizoid PT Score					
				95% CI						95% CI		
	B	SE	z	LL	UL	*p*	B	SE	z	LL	UL	*p*
HARS Anxiety as outcome	r^2^ = 0.56						r^2^ = 0.56					
PT ABS II HRAS	0.01	0.03	0.32	−0.04	0.06	0.75	−0.00	0.01	−0.04	−0.03	0.02	0.97
PT DAS-A HRAS	0.02	0.05	0.48	−0.07	0.12	0.63	0.03	0.04	0.89	−0.04	0.11	0.37
PT ATQ HRAS	0.20	0.09	2.11	0.01	0.38	0.04	0.15	0.09	1.73	−0.02	0.31	0.08
Direct effect	0.40	0.15	2.64	0.10	0.69	0.01	0.42	0.14	3.09	0.16	0.69	0.002
Total indirect	0.23	0.11	2.11	0.02	0.44	0.04	0.18	0.09	1.92	0.00	0.36	0.06
Total effect	0.63	0.15	4.08	0.33	0.93	<0.001	0.60	0.15	3.94	0.30	0.90	<0.001
BDI-II Depression as outcome	r^2^ = 0.56						r^2^ = 0.54					
PT ABS II BDI-II	0.06	0.06	1.16	−0.04	0.17	0.25	0.03	0.05	0.62	−0.07	0.12	0.54
PT DAS-A BDI-II	0.01	0.05	0.23	−0.08	0.10	0.82	0.02	0.03	0.67	−0.03	0.07	0.50
PT ATQ BDI-II	0.23	0.10	2.22	0.03	0.43	0.03	0.18	0.10	1.79	−0.02	0.37	0.07
Direct effect	0.22	0.14	1.55	−0.06	0.51	0.12	0.24	0.13	1.83	−0.02	0.50	0.07
Total indirect	0.31	0.13	2.41	0.06	0.55	0.02	0.22	0.11	1.99	0.003	0.45	0.05
Total effect	0.53	0.16	3.23	0.21	0.85	0.001	0.47	0.16	2.87	0.15	0.79	0.004
CFS Fatigue as outcome	r^2^ = 0.67						r^2^ = 0.60					
PT ABS II CFS	−0.01	0.02	−0.51	−0.04	0.03	0.61	−0.01	0.02	−0.58	−0.05	0.03	0.56
PT DAS-A CFS	−0.05	0.04	−1.39	−0.12	0.02	0.17	−0.001	0.02	−0.04	−0.03	0.03	0.97
PT ATQ CFS	0.19	0.08	2.31	0.03	0.36	0.02	0.16	0.08	1.84	−0.01	0.32	0.07
Direct effect	0.40	0.10	4.19	0.21	0.58	<0.001	0.29	0.10	3.00	0.10	0.48	0.003
Total indirect	0.13	0.09	1.42	−0.05	0.31	0.16	0.14	0.09	1.62	−0.03	0.32	0.11
Total effect	0.53	0.12	4.59	0.30	0.75	<0.001	0.43	0.12	3.52	0.19	0.67	<0.001
EQ-5D-3L health status as outcome	r^2^ = 0.72						r^2^ = 0.70					
PT ABS II EQ-5D-3L	0.001	0.004	0.13	−0.01	0.01	0.89	−0.001	0.003	−0.42	−0.01	0.004	0.67
PT DAS-A EQ-5D-3L	−0.02	0.01	−1.77	−0.04	0.002	0.08	−0.01	0.01	−0.92	−0.02	0.01	0.36
PT ATQ EQ-5D-3L	0.04	0.02	2.29	0.01	0.08	0.02	0.04	0.02	1.85	0.00	0.08	0.07
Direct effect	0.05	0.02	1.97	0.00	0.09	0.05	−0.00	0.02	−0.02	−0.04	0.04	0.99
Total indirect	0.03	0.02	1.12	−0.02	0.07	0.26	0.03	0.02	1.42	−0.01	0.08	0.16
Total effect	0.07	0.03	2.53	0.02	0.13	0.01	0.03	0.03	1.05	−0.03	0.09	0.30

Note. r squared for the total effect; B—unstandardized regression coefficient, SE—standard error, z—z-value, *p*—*p*-value.

## Data Availability

The data presented in this study are available upon request from the corresponding author (cosmin.popa@umfst.ro).
